# Adaptive Optimization and Dynamic Representation Method for Asynchronous Data Based on Regional Correlation Degree

**DOI:** 10.3390/s24237430

**Published:** 2024-11-21

**Authors:** Sichao Tang, Yuchen Zhao, Hengyi Lv, Ming Sun, Yang Feng, Zeshu Zhang

**Affiliations:** 1 Changchun Institute of Optics, Fine Mechanics and Physics, Chinese Academy of Sciences, Changchun 130033, China; tangsichao21@mails.ucas.ac.cn (S.T.); lvhengyi@ciomp.ac.cn (H.L.); sunming@ciomp.ac.cn (M.S.); fengyang16@mails.ucas.edu.cn (Y.F.); zhangzeshu@ciomp.ac.cn (Z.Z.); 2 University of Chinese Academy of Sciences, Beijing 100049, China

**Keywords:** event cameras, slicing methods, event representations

## Abstract

Event cameras, as bio-inspired visual sensors, offer significant advantages in their high dynamic range and high temporal resolution for visual tasks. These capabilities enable efficient and reliable motion estimation even in the most complex scenes. However, these advantages come with certain trade-offs. For instance, current event-based vision sensors have low spatial resolution, and the process of event representation can result in varying degrees of data redundancy and incompleteness. Additionally, due to the inherent characteristics of event stream data, they cannot be utilized directly; pre-processing steps such as slicing and frame compression are required. Currently, various pre-processing algorithms exist for slicing and compressing event streams. However, these methods fall short when dealing with multiple subjects moving at different and varying speeds within the event stream, potentially exacerbating the inherent deficiencies of the event information flow. To address this longstanding issue, we propose a novel and efficient Asynchronous Spike Dynamic Metric and Slicing algorithm (ASDMS). ASDMS adaptively segments the event stream into fragments of varying lengths based on the spatiotemporal structure and polarity attributes of the events. Moreover, we introduce a new Adaptive Spatiotemporal Subject Surface Compensation algorithm (ASSSC). ASSSC compensates for missing motion information in the event stream and removes redundant information, thereby achieving better performance and effectiveness in event stream segmentation compared to existing event representation algorithms. Additionally, after compressing the processed results into frame images, the imaging quality is significantly improved. Finally, we propose a new evaluation metric, the Actual Performance Efficiency Discrepancy (APED), which combines actual distortion rate and event information entropy to quantify and compare the effectiveness of our method against other existing event representation methods. The final experimental results demonstrate that our event representation method outperforms existing approaches and addresses the shortcomings of current methods in handling event streams with multiple entities moving at varying speeds simultaneously.

## 1. Introduction

**Figure 1:**
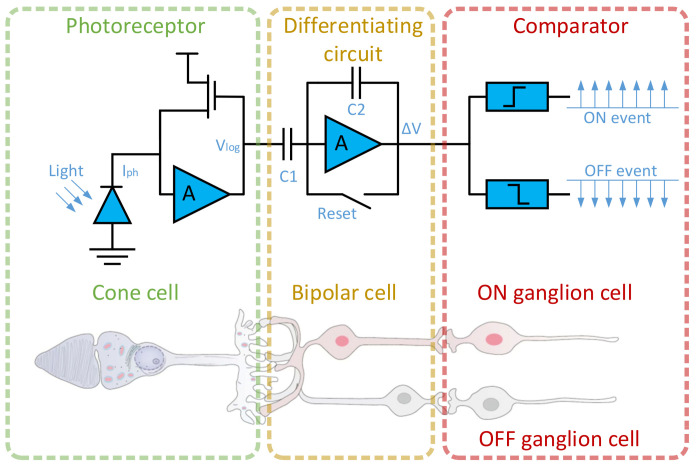
Schematic diagram of the human retina model and corresponding event camera pixel circuit.

Event cameras (such as DVS [1], DAVIS [2], and ATIS [3]) are bio-inspired visual sensors, with their internal structure illustrated in Figure 1. Unlike traditional cameras, event cameras do not capture images at a fixed frame rate. Instead, each pixel independently and asynchronously responds to changes in intensity within the environment. Due to their asynchronous nature, similar to the biological retina, they can accurately and efficiently capture motion information in natural scenes [4,5], particularly movements caused by dynamic objects [6,7]. This asynchronous characteristic makes event cameras well-suited for a wide range of applications, including target tracking, robotics, motion estimation, autonomous vehicles, and virtual reality. As depicted in Figure 1, the structure of an event camera enables each pixel to generate an “on” event, which includes the pixel’s coordinates and the current timestamp, when there is an increase in luminance at the corresponding location. Conversely, if the luminance decreases, the event camera records an “off” event with the pixel’s coordinates and the current timestamp. This binary event-based output requires specialized processing techniques to reconstruct meaningful visual information from the asynchronous event data.

Specifically, a spike will be fired from a pixel $u_{n}=\left[x_{n},y_{n}\right]$ at the time $t_{n}$ when the intensity change reaches a firing threshold $C_{\mathrm{th}\;}$, and the definition is as follows.

(1) $$\Delta \mathrm{lnL}≐\mathrm{lnL}\left(u_{n},t_{n}\right)-\mathrm{lnL}\left(u_{n},t_{n}-{\Delta t}_{n}\right)=p_{n}C_{\mathrm{th}}.$$

The spike is defined as $e_{n}=\left(x_{n},y_{n},t_{n},p_{n}\right)$. ${\Delta t}_{n}$ is the time since the last spike at the same pixel, and the polarity $p_{n}\in \left\{1,-1\right\}$ respectively represents ON or OFF spikes. In event cameras, a sequence of ordered spike firing timestamps for each pixel can be defined as a spike train $A=\left\{t_{n}\in \Gamma :n=1,\ldots ,N\right\}$, and its mathematical expression can be presented as follows:

(2) $$A\left(t\right)=\sum_{n=1}^{N}p_{n}\delta \left(t-t_{n}\right),$$

where N is the number of spikes in a single pixel during the time interval Γ, and δ(⋅) refers to the Dirac delta function, with δ(t)=0∀x≠0 and ∫δ(t)dt=1. Similarly, the asynchronous spike stream $S=\left\{x_{n},y_{n},t_{n}\in \Gamma_{s}:n=1,\ldots ,N\right\}$, which represents the event information, generated by pixels in the spatiotemporal interval $\Gamma_{s}$ can be divided into event cuboids [8,9] s∈S. Its corresponding mathematical formula is as follows.

(3) $$S\left(x,y,t\right)=\sum_{n=1}^{N}p_{n}\delta \left(x-x_{n},y-y_{n},t-t_{n}\right).$$

The output data of an event camera consist of a series of events that encode the time, location, and polarity information of brightness changes at specific pixel points. While this provides an advantage for event cameras, it also means that each event individually carries very little information about the scene. Therefore, before interpreting or utilizing the information from the event stream, we need to aggregate the data for further processing. In the field of event-based vision, existing representation methods can be broadly classified into two categories: sparse representation and dense representation. Sparse representation methods [10,11,12,13] preserve the sparsity of events, but current hardware and subsequent data processing algorithms are not mature or targeted enough. Consequently, event data preprocessed using these methods cannot be extended to more complex tasks. Specifically, the sparse event data required by asynchronous pulse neural networks [10,11,13] are limited by the lack of specialized hardware and efficient backpropagation algorithms. Moreover, point cloud encoders [14,15,16] are essential for handling the spatiotemporal characteristics of event data, but they incur high computational costs and result in significant noise. Graph neural networks [17,18,19,20,21,22] offer considerable scalability and can achieve high performance in most vision tasks, but their accuracy in event-based vision is still inferior to dense methods.

Conversely, dense representation methods [23,24,25,26] provide better performance, as they can integrate with existing advanced machine learning algorithms and neural network architectures. Early dense representation methods mostly converted events into histograms [23], time surfaces [24], or a combination of both, followed by standard image processing algorithm models for subsequent processing. However, these methods typically used only a few channels, capturing only low-dimensional representations of the events. Further research attempted to capture more event information by computing higher-order moments [27] or stacking multiple time windows of events [25]. Nonetheless, these methods inherently rely on fixed time windows for event stacking, leading to issues when event rates become too high or too low. To address these shortcomings, event count-based stacking methods [26] were developed. Concurrently, a biologically inspired method introduced the use of Time-Ordered Recent Event (TORE) [28], which aggregates events into queues. However, their efficiency was relatively poor, similar to existing voxel grid [25] representation methods. Recently, Nam et al. proposed a more efficient representation method [29], which divides events into multiple overlapping windows, halving the number of events at each stage and enhancing robustness in processing data from different motion scenes.

However, due to the inherent sparsity and asynchronicity of event cameras, classical computer vision algorithms, although effective, are not fully applicable. To leverage the spatiotemporal structural characteristics and polarity attributes of the event stream, we propose a Dynamic Asynchronous Data Metric and Slicing algorithm (ASDMS). This algorithm first divides the event stream into main and non-main event streams. The “main” event stream consists of events with higher significance based on certain criteria, while the “non-main” stream contains the remaining events. ASDMS then segments these streams into event cuboids, which are spatiotemporal volumes of a specified size, based on a default time span. The polarity of events within each event cuboid is used as the basis for calculating overall density, and this new event density is introduced into the time window as a reference to dynamically adjust the length of the event cuboids. After adjusting the lengths of the event cuboids in both event streams, the cuboids are correspondingly matched and merged into a new, modified event stream. The ASDMS algorithm, upon adjusting the cuboid spans and outputting the new event stream, may cause loss and the redundancy of events within each cuboid. To address this, we define a new Adaptive Spatiotemporal Subject Surface Compensation algorithm (ASSSC). ASSSC uses the main event stream as a base to minimize the impact of redundant, low-correlation non-main events surrounding the main spatial area in the new event stream. It then selects highly correlated events to compensate for the missing main events within the cuboids. This approach not only effectively addresses the issue of poor slicing performance when dealing with multiple objects moving at significantly different speeds, as mentioned at the end of article [30], but also retains more information in the preprocessed data.

Lastly, we need to quantitatively evaluate our method against existing methods to provide an intuitive comparison. Few papers have studied the performance of event stream data tasks. Although [31,32] presented small-scale studies on selecting event representations, only Gehrig et al. [33] conducted a large-scale investigation of event representations by studying various inputs across multiple tasks. They explored the advantages of splitting polarity and incorporating timestamps into event representations. However, single-task studies still have high computational performance requirements, limiting the number of representations that can be compared. Consequently, their study did not cover many event representations, particularly those considering different window sizes as in later works [26,29] or more advanced aggregation and measurement as in [27]. Moreover, existing comparison methods do not adequately quantify the degree of distortion in the final results due to the deletion of crucial distinguishing features during event representation, nor the loss of the main information-carrying parts. To address these shortcomings, we propose a method for quickly comparing event representations, bypassing the need for pre-training neural networks. This method combines prior knowledge of Gromov–Wasserstein Discrepancy [34] and information entropy [35], directly calculating the Actual Performance Efficiency Discrepancy (APED) between the raw events and their representations. A lower APED indicates a lower actual distortion rate and higher retention of main information-carrying events. Our contributions include the following three main points:

- We propose a Dynamic Asynchronous Data Metric and Slicing algorithm (ASDMS) that dynamically adjusts the slicing span of events based on the spatiotemporal structure and polarity information of the event stream;
- We introduce an Adaptive Spatiotemporal Subject Surface Compensation algorithm (ASSSC) that repairs the main information-carrying parts of the new event stream after slicing based on the correlation between main and overall events, removing redundant events in the spatiotemporal correlation area;
- We propose a new evaluation metric, Actual Performance Efficiency Discrepancy (APED), which quantifies the effectiveness of each representation method in handling the primary information-carrying events in the event stream.

## 2. Materials and Methods

### 2.1. Asynchronous Spike Dynamic Metric and Slicing Algorithm

Before performing the slicing operation, we use the previously proposed MPAR method [36] to divide the original event stream into main event stream $S_{i}$ and non-main event stream $S_{j}$ based on overall correlation. This allows the subsequent slicing process to obtain dynamic parameters that better represent the interrelationship between the data. In the following algorithm process, we will measure and segment the event stream from the perspectives of spatiotemporal structure and polarity attributes.

Nowadays, some researchers have introduced computational structures to provide a theoretical foundation for measuring asynchronous event stream data [37,38,39]. Based on these results, we have equipped the previously mentioned asynchronous spike data stream S, which represents event information, with a new metric d and proposed a new complete and separable metric space (S,d). When S is a compact subset equipped with Euclidean distance, S is also referred to as a locally finite configuration. Therefore, $S\left(x,y,t\right)=\sum_{n=1}^{N}p_{n}\delta \left(x-x_{n},y-y_{n},t-t_{n}\right),0<N<\infty$, and this denotes a measurable mapping from some probability space [Φ,Ω,θ] to a measurable space. If N<∞, then S represents marked spatiotemporal points (MSPs) in a finite space. Probability space [Φ,Ω,θ] is the mathematical model of a random experiment, where sample space Φ is the set of all possible outcomes. Ω is the σ-algebra of subsets of the sample space. When 0≪Ω≪1, θ represents the probability measure.

Quantifying asynchronous spikes is a challenging process due to the lack of standard algebraic operations. Kernel methods [37,40,41] offer a general framework for measuring spike sequences. These methods can extend linear modeling in the input space to nonlinear modeling, specifically mapping abstract objects to Hilbert space. This characteristic provides a new approach to addressing key issues in the quantification process. Therefore, we choose to use Gaussian kernel-based methods to measure the distance between asynchronous spikes in MSPs. We let $s_{i}$ and $s_{j}$ be two event cuboids in the spatiotemporal interval $\Gamma_{s}$, respectively, and the inner product is introduced to measure the distance between asynchronous spikes in a Hilbert space in Formula (4).

(4) $$∥s_{i}-s_{j}∥≜\sqrt{\kappa \left(s_{i},s_{i}\right)+\kappa \left(s_{j},s_{j}\right)-2\kappa \left(s_{i},s_{j}\right)},$$

where $\kappa \left(s_{i},s_{j}\right)$ is the inner product of $s_{i}$ and $s_{j}$.

**Figure 2:**
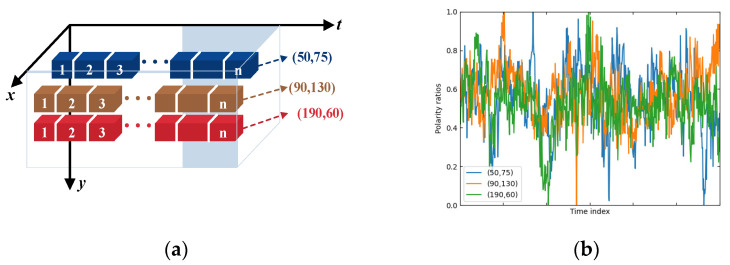
(**a**) We consider the light intensity change signals received by the corresponding pixels as computational elements in the time domain. (**b**) From the statistical results, it can be seen that the ON polarity ratio varies randomly over the time index.

When analyzing the polarity attributes of event streams, we found that existing spike sequence measurement methods [40,42,43,44] rarely utilize polarity attributes. Therefore, we adopted the approach of describing pulse data using conditional probability density functions as seen in [37,45]. As shown in Figure 2a, we selected three representative pixel coordinates from the event camera as the origin of the spike flow’s vertical axis. The distance between spike trains is calculated using a single neuron spike train metric and increases with the pixel index. The measurement results, as shown in Figure 2b, indicate that the ON and OFF polarity ratios vary randomly. Consequently, we can use the polarity attribute as a prior probability distribution in spike metrics, describing MSPs using a conditional intensity function. This approach integrates polarity attributes as a key calculation point in the algorithm process.

The expression of the conditional strength function is as follows:

(5) $$\lambda \left(x,y,t,p|H_{t}\right)=\frac{f\left(x,y,t,p|H_{t}\right)}{1-F\left(x,y,t|H_{t}\right)},$$

(6) $$H_{t}=\left\{e_{n}\in \Gamma_{s}|t_{n}<t\right\},$$

where $F\left(x,y,t|H_{t}\right)$ is the cumulative distribution function, and $f\left(x,y,t,p|H_{t}\right)$ is the conditional probability density function in spiking history $H_{t}$.

Next, by applying Bayes’ theorem to derive Formula (5), we obtain the following new expression for the conditional intensity function.

(7) $$\begin{array}{rr} \lambda \left(x,y,t,p|H_{t}\right) & =\frac{f\left(x,y,t|H_{t}\right)}{1-F\left(x,y,t|H_{t}\right)}\cdot f\left(p|H_{t},x,y,t\right) \end{array}=\lambda \left(x,y,t|H_{t}\right)\cdot f\left(p|H_{t},x,y,t\right),$$

where $\sum_{p\in \left\{1,-1\right\}}\iint_{\Gamma_{s}}\lambda^{2}\left(x,y,t,p|H_{t}\right)\mathrm{dxdydt}<\infty$; therefore, $\lambda \left(x,y,t,p|H_{t}\right)$ is an element of $L_{2}\left(\Gamma_{s}\right)$ space.

Next, we choose to use the smoothing function h(x,y,t) to capture the spatiotemporal structure and use 3D convolution to convert discrete spikes into continuous intensity functions. It is similar to the conditional strength function in history time $H_{t}$ without considering the polarity, and it is computed as follows:

(8) $$\begin{array}{rr} \lambda \left(x,y,t|H_{t}\right) & =s\left(x,y,t\right)*h\left(x,y,t\right) \end{array}=\sum_{n=1}^{N}h\left(x-x_{n},y-y_{n},t-t_{n}\right).$$

Then, $f\left(p|H_{t},x,y,t\right)$ can be calculated based on the polarity probability distribution in history time $H_{t}$, and it is modeled as:

(9) $$f\left(p|H_{t},x,y,t\right)=\frac{\mathrm{Nu}\left\{e_{n}\in \Gamma_{s}|p_{n}=p,x_{n}<x,y_{n}<y,t_{n}<t\right\}}{\mathrm{Nu}\left\{e_{n}\in \Gamma_{s}\right\}},$$

where Nu{} represents the counting result of event numbers in the spatiotemporal interval $\Gamma_{s}$. Additionally, for any two event cuboids $s_{i}$ and $s_{j}$, the inner product $\kappa \left(s_{i},s_{j}\right)$ can be given by Formula (10).

(10) $$\begin{array}{cc} \kappa \left(s_{i},s_{j}\right) & ={\left\{\lambda_{s_{i}}\left(x,y,t,p|H_{t}\right),\lambda_{s_{j}}\left(x,y,t,p|H_{t}\right)\right\}}_{L_{2}\left(\Gamma_{s}\right)}\\ & =\underset{p\in \left\{-1,1\right\}}{\sum }\int \int \int_{\Gamma_{s}}\left(\lambda_{s_{i}}\cdot \lambda_{s_{j}}\right)\mathrm{dxdydt}. \end{array}$$

The formulas substituted into (7) and (8) can transform Formula (10) into the following form:

(11) $$\begin{array}{rr} \kappa \left(s_{i},s_{j}\right)= & \;\underset{p\in \left\{-1,1\right\}}{\sum }\overset{N_{i}}{\underset{m=1}{\sum }}\overset{N_{j}}{\underset{n=1}{\sum }}\int \int \int_{\Gamma_{s}}f_{s}\left(p|H_{t},x,y,t\right)\cdot H\left(x,y,t\right)\mathrm{dxdydt} \end{array},$$

(12) $$f_{s}\left(p|H_{t},x,y,t\right)=f_{s_{i}}\left(p|H_{t},x,y,t\right)\cdot f_{s_{j}}\left(p|H_{t},x,y,t\right),$$

(13) $$H\left(x,y,t\right)=h\left(x-x_{m}^{\left(i\right)},y-y_{m}^{\left(i\right)},t-t_{m}^{\left(i\right)}\right)\cdot h\left(x-x_{n}^{\left(j\right)},y-y_{n}^{\left(j\right)},t-t_{n}^{\left(j\right)}\right),$$

where $N_{i}$ and $N_{j}$ are the number of events in event cuboids $s_{i}$ and $s_{j}$.

For two spikes of events $e_{m}^{\left(i\right)}$ and $e_{n}^{\left(j\right)}$ in event cuboids $s_{i}$ and $s_{j}$, respectively, the inner product between two spikes can be represented as follows:

(14) $$\begin{array}{rr} \kappa \left(e_{m}^{\left(i\right)},e_{n}^{\left(j\right)}\right)=\int \int \int_{\Gamma_{s}}H\left(x,y,t\right) & \mathrm{dxdydt} \end{array}.$$

When processing the calculation of inner product, we use Gaussian kernel as the smoothing function and define it as follows:

(15) $$h\left(x,y,t\right)=\frac{e^{-\frac{x^{2}}{2\sigma_{x}^{2}}}}{l_{x}}\cdot \frac{e^{-\frac{y^{2}}{2\sigma_{y}^{2}}}}{l_{y}}\cdot \frac{e^{-\frac{t^{2}}{2\sigma_{t}^{2}}}}{l_{t}},$$

where $\sigma_{x},\sigma_{y}$, and $\sigma_{t}$ are the standard deviation parameters of the Gaussian kernel, and $l_{x}={\left(\sqrt{\pi }\sigma_{x}\right)}^{1/2},l_{y}=$ ${\left(\sqrt{\pi }\sigma_{y}\right)}^{1/2}$, and $l_{t}={\left(\sqrt{\pi }\sigma_{t}\right)}^{1/2}$. So, (14) can be re-expressed as follows:

(16) $$\kappa \left(e_{m}^{\left(i\right)},e_{n}^{\left(j\right)}\right)=e^{-\frac{{\left(x_{m}^{\left(i\right)}-x_{n}^{\left(j\right)}\right)}^{2}}{4\sigma_{x}^{2}}-\frac{{\left(y_{m}^{\left(i\right)}-y_{n}^{\left(j\right)}\right)}^{2}}{4\sigma_{y}^{2}}-\frac{{\left(t_{m}^{\left(i\right)}-t_{n}^{\left(j\right)}\right)}^{2}}{4\sigma_{t}^{2}}}.$$

In summary, the inner product between two event cuboids can be further rewritten as follows:

(17) $$\kappa \left(s_{i},s_{j}\right)=\sum_{m=1}^{N_{i}}\sum_{n=1}^{N_{j}}\kappa \left(e_{m}^{\left(i\right)},e_{n}^{\left(j\right)}\right)\cdot \underset{p\in \left\{-1,1\right\}}{\sum }f_{s_{i}}\left(p|H_{t},x,y,t\right)\cdot f_{s_{j}}\left(p|H_{t},x,y,t\right),$$

(18) $$R\left(s_{i},s_{j}\right)=\sum_{p\in \left\{1,-1\right\}}f_{s_{i}}\left(p|H_{t},x,y,t\right)f_{s_{j}}\left(p|H_{t},x,y,t\right),$$

Here, $R\left(s_{i},s_{j}\right)$ represents the relative polarity static in the spike cube. Generally, the optimal kernel parameter $\theta =\left\{\sigma_{x},\sigma_{y},\sigma_{t}\right\}$ of the three-dimensional Gaussian kernel function involved in Formula (15) can be continuously optimized by minimizing the fitting error [46]. To obtain more accurate calculation results, we refer to the parameter structure of compression ratio [40,47] and the definition of PSNR [48,49], defining PSNR as the performance score $p_{s}$. The performance score might measure the algorithm’s efficiency in maintaining information fidelity, like minimizing motion blur, preserving object details, or reducing data redundancy. It could also be a comparative measure, assessing the balance between computational cost and accuracy of the event data’s representation for specific tasks, like detection, tracking, or segmentation. Based on this, we calculate the correlation coefficient between the spike metric distance d and the performance score.

Therefore, the error function J(θ) corresponding to the optimal kernel function is defined as follows:

(19) $$J\left(\theta \right)=\sum_{i\in R}\sum_{j\in D}{∥d\left(S_{i},S_{j},\theta \right)-f_{b}\left(p_{s}\left(S_{i},S_{j}\right),b\right)∥}^{2}+\gamma ∥b∥.$$

where d is the distance between two spike streams. R and D are, respectively, the corresponding sets. $f_{b}$ is a polynomial function of degree b, which is able to fit curve between the distance d and performance score $p_{s}$. γ is a hyperparameter that weights the relative contribution of the norm penalty term.

Next, as shown in Formulas (20) and (21), we use the gradient method to iterate the kernel parameters in order to minimize the error.

(20) $$\theta^{\left(n+1\right)}=\theta^{\left(n\right)}-\eta \cdot \nabla \frac{\partial J}{\partial \theta }.$$

(21) $$\theta^{*}=\underset{\theta }{\mathrm{argmin}}\;J\left(\theta \right).$$

The learning rate η is a hyperparameter that determines how we adjust the loss gradient. We set the initial values for the learning rate η and the maximum number of iterations $N_{o}$ and use ε to represent a positive number approaching infinity. We then fit a polynomial function $f_{b}\left(p_{s}\left(S_{i},S_{j},\theta^{\left(0\right)}\right),b\right)$ between the distance and performance score, using Formula (17) to update the kernel parameter $\theta^{\left(n\right)}$, where n represents the iteration step, initially set to 0. The updating process stops when J(θ)<ε or $n\geq N_{o}$, at which point we have achieved the minimum error. Otherwise, we increment n by one and continue iterating. Next, we need to calculate the distance relationship between the event cuboid and its corresponding event stream, with the detailed process described in Algorithm 1, where learning rate η is a hyperparameter that determines how much we adjust θ with respect to the loss gradient.
**Algorithm 1. Asynchronous Spike Dynamic Metric and Slicing.**$\mathrm{Input}:\;\mathrm{Two}\;\mathrm{spike}\;\mathrm{event}\;\mathrm{streams}\;S_{i}{\;\mathrm{and}\;S}_{j}$ $\mathrm{Output}:\;\mathrm{The}\;\mathrm{distance}\;∥S_{i}-S_{j}∥\;\mathrm{between}\;\mathrm{two}\;\mathrm{streams}$ $1\;\;\;\mathrm{Divide}\;\mathrm{streams}\;S_{i}{\;\mathrm{and}\;S}_{j}{\;\mathrm{into}\;K\;\mathrm{spike}\;\mathrm{cuboids}\;s}_{i}^{k}{\;\mathrm{and}\;s}_{j}^{k}$ 2   **For** k =1, 2 …, K **do** $3\;\;\;\;\;\;\;\;\;\;\;\mathrm{Calculate}\;\mathrm{the}\;f_{s_{i}}^{k}{\;\mathrm{and}\;f}_{s_{j}}^{k}\;\mathrm{based}\;\mathrm{on}\;\mathrm{formula}\;\left(8\right)\;\mathrm{and}\;\mathrm{polarity}\;\mathrm{of}\;\left(s_{i}^{k},s_{j}^{k}\right)$ $4\;\;\;\;\;\;\;\;\;\;\;\;\mathrm{Compute}\;\mathrm{the}\;\mathrm{relative}\;\mathrm{polarity}\;\mathrm{statics}\;R\left(s_{i},s_{j}\right)\;\mathrm{based}\;\mathrm{on}\;\mathrm{Formula}\;\left(16\right)$ $5\;\;\;\;\;\;\;\;\;\;\;\;\mathrm{Obtain}\;\mathrm{inner}\;\mathrm{product}\;\kappa \left(s_{i},s_{j}\right)\;\mathrm{of}\;(s_{i}^{k},s_{j}^{k})\;\mathrm{based}\;\mathrm{on}\;\mathrm{Formula}\;\left(15\right)$ $6\;\;\;\;\;\;\;\mathrm{Obtain}\;\mathrm{the}\;\mathrm{distance}\;∥s_{i}^{k}-s_{j}^{k}∥\;\mathrm{of}\;\mathrm{spike}\;\mathrm{cuboids}\;\mathrm{based}\;\mathrm{on}\;\mathrm{Formula}\;\left(4\right)$ $7\;\;\;\;\;\;\;\mathrm{Obtain}\;\mathrm{the}\;\mathrm{value}\;\mathrm{of}\;∥S_{i}-S_{j}∥\;\mathrm{by}\;\mathrm{accumulating}\;∥s_{i}^{k}-s_{j}^{k}∥$ 8   **End for** $9\;\;\;\mathrm{Return}\;∥S_{i}-S_{j}∥$

**Figure 3:**
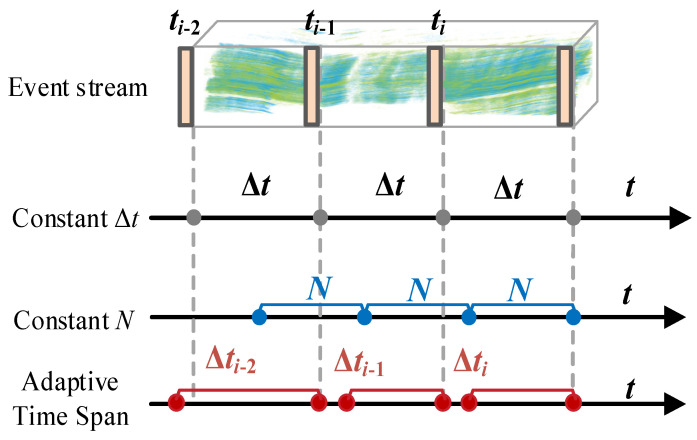
This graph represents the time span changes of each event cuboid processed by our algorithm.

The obtained calculation results are substituted into the following adaptive time span calculation formula:

(22) $$\Delta {t_{i}}^{*}=\underset{\Delta t_{i}}{\arg\;\min}\;|\frac{∥S_{i}∥}{∥S_{i}-S_{j}∥}-\frac{\theta^{\left(i\right)}\left(\Delta t_{i-1}-\Delta t_{i}\right)}{\left|\theta^{\left(i-1\right)}-\theta^{\left(i\right)}\right|}\mathrm{Nu}\left\{e_{n}\in \Gamma_{s}\right\}|,$$

(23) $$\Delta {t_{j}}^{*}=\underset{\Delta t_{j}}{\arg\;\min}\;|\frac{∥S_{j}∥}{∥S_{i}-S_{j}∥}-\frac{\theta^{\left(j\right)}\left(\Delta t_{j-1}-\Delta t_{j}\right)}{\left|\theta^{\left(j-1\right)}-\theta^{\left(j\right)}\right|}\mathrm{Nu}\left\{e_{n}\in \Gamma_{s}\right\}|,$$

where Δt represents the time span of the event cuboid in microseconds. This allows us to segment a continuous event stream into intervals of varying lengths based on time labels. These intervals can be discontinuous or overlapping. The dynamically sliced event stream is illustrated in Figure 3. Here, the time label interval is the base time divided by the time window, with a label assigned every 5000 microseconds. After re-slicing both the main and non-main event streams, the event cuboids in both streams are aligned and merged based on the time labels. In this way, we minimize the occurrence of redundancy in each event cuboid after being compressed into frames. However, as a result, we also lose some subject-related events to a certain extent. In the next section, we will compensate for the subjects appearing in all cuboids based on the two processed event streams.

### 2.2. Adaptive Spatiotemporal Subject Surface Compensation Algorithm

To describe the influence of different events on each other over time, we refer to the definitions of event relationships in [50,51], introducing the concept of the time surface to describe the local spatiotemporal surroundings of related events. The time surface can be transformed into a local spatial operator acting on the i-th event in the main event stream, defined as follows:

(24) $$T_{s_{i}}\left(z,q\right)=\{\begin{array}{ll} e^{-\frac{t_{i}-t\left(x_{i}+z,y_{i}+z,q\right)}{\tau }} & {\mathrm{if}\;p}_{i}=q\\ 0 & \mathrm{otherwise} \end{array},$$

(25) $$\left(z,q\right)\in {\left[-\rho ,\rho \right]}^{2}\times \{-1,1\},$$

where ρ is the radius of the spatial neighborhood used to calculate the time surface, and $t\left(x_{i}+z,y_{i}+z,q\right)$ represents the timestamp of the last event with polarity q received from pixel location $\left(x_{i}+z,y_{i}+z\right)$. τ means a decay factor that gives less weight to events farther in the past. Intuitively, the time surface encodes the asynchronous information in a neighborhood of events, providing temporal and spatial relationships between events.

**Figure 4:**
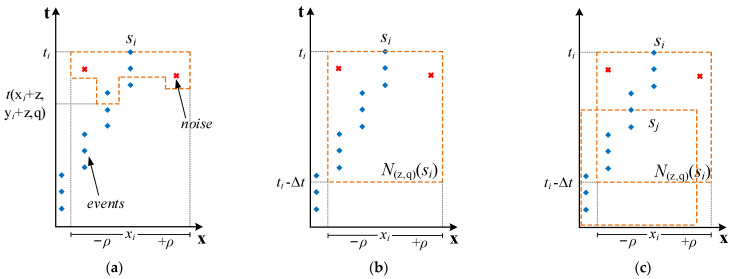
This figure illustrates the time surface of events in the original event stream. For clarity, only the x–t components are shown. Red crosses represent non-main events, and blue dots represent main events. (**a**) In the time surface described in [50] (corresponding to Formula (24)), only the occurrence frequency of the nearest events around the main event is considered. Consequently, non-main events with disruptive effects may have significant weight. (**b**) The local memory time surface corresponding to Formula (26) considers the influence weight of historical events within the current spatiotemporal window. This approach reduces the ratio of non-main events involved in the time surface calculation, better capturing the true dynamics of the event stream. (**c**) By spatially averaging the time surfaces of all events in adjacent cells, the time surface corresponding to Formula (29) can be further regularized. Due to the spatiotemporal regularization, the influence of non-main events is almost completely suppressed.

In order to construct the required feature event representation, we need to first generalize the time plane proposed above. As shown in Figure 4a, the time surface of Formula (24) only uses the time $t\left(x_{i}+z,y_{i}+z,q\right)$ of the last event received near the pixel on the time surface, resulting in the descriptor being overly sensitive to minor changes in non-main events or the original event stream.

To address this shortcoming, we use historical events within the time window Δt adjusted by the ASDMS algorithm from the previous section to calculate the time surface. More precisely, we define a local memory time surface as follows:

(26) $$T_{s_{i}}\left(z,q\right)=\{\begin{array}{ll} \underset{s_{j}\in N_{\left(z,q\right)}\left(s_{i}\right)}{\sum }e^{-\frac{t_{i}-t_{j}}{\tau }} & {\mathrm{if}\;p}_{i}=q\\ 0 & \mathrm{otherwise} \end{array},$$

(27) $$N_{\left(z,q\right)}\left(s_{i}\right)=\left\{s_{j}:x_{j}=x_{i}+z,y_{j}=y_{i}+z,t_{j}\in \left(t_{i}-\Delta t,t_{i}\right),p_{j}=q\right\}.$$

As shown in Figure 4b, Formula (26) more effectively mitigates the impact of non-main events and minor variations in the event stream, while robustly describing the actual dynamics occurring in the captured scene. Next, we refer to the approach in [52] for obtaining invariance in speed and contrast, using the local memory time surface as a basic operator and grouping adjacent pixels into cells of equal size and quantity ${\left\{C_{l}\right\}}_{l=1}^{L}$. For each cell, we sum the time surface components of the events within it into a histogram as follows:

(28) $$h_{C}^{0}\left(z,p\right)=\sum_{s_{i}\in C}T_{s_{i}}\left(z,p\right),$$

where $s_{i}\in C$ represents the pixel coordinates of the current event within the cell range. Since the event camera sensor generates more events for high-contrast objects compared to low-contrast objects, we normalize $h^{0}$ by the number of events within the spatiotemporal window to make the descriptor of the cell more contrastive. The histogram definition of the event group cells is as follows:

(29) $$h_{C}\left(z,p\right)=\frac{1}{\left|C\right|}h_{C}^{0}\left(z,p\right)=\frac{1}{\left|C\right|}\sum_{s_{i}\in C}T_{s_{i}}\left(z,p\right).$$

The schematic of the cell histogram is shown in Figure 4c. This approach regularizes space and time, reducing the influence of non-main events on the main events in the new event stream. Next, we need to compensate for the loss of main events containing important information due to the change in time span in the previous algorithm.

The events within time segment C and cell C{x,y,t} are represented by triplet 66. Referring to [52], we use a warp field $\Phi \left(x,y,t-t_{i}\right):\left(x,y,t\right)\to \left(x^{\prime },y^{\prime },t\right)$ to represent the planar displacement that maps events at time t to their positions at time $t_{i}$. The goal is to find a corresponding motion compensation warp field $\Phi :R^{3}\to R^{3}$ that ensures the events used to supplement the event stream have the most appropriate density when projected onto the image plane. We define these primary compensation events as follows:

(30) $$C^{\prime }=\Pi \left\{\Phi \left(U\right)\right\}=\Pi \left\{\Phi \left(x,y,t-t_{i}\right)\right\}=\left\{x^{\prime },y^{\prime },0\right\},$$

(31) ∀{x,y,t}∈C,

where Π represents the time projection function that projects primary compensation events along the time axis, and Π can be used to reduce the data’s dimensionality: $R^{3}\to R^{2}$. Regarding the geometric properties presented by the event stream, the warp field encodes information for each event. Thus, we use two discrete mappings that encode the intensity and temporal attributes of the event stream to represent the available data in C. These are denoted as Υ and Ψ.

To calculate the event density D of the primary compensation events $C^{\prime }$, we discretize the image plane into pixel regions of specified size. We use the symbol $\left(m,n\right)\in N^{2}$ to represent integer pixel coordinates and $\left(x^{\prime },y^{\prime },t\right)\in R^{3}$ to represent the coordinates of events after displacement. Each projected event in $C^{\prime }$ is mapped to a discrete pixel, with the total number of events mapped to that pixel recorded as its value as follows:

(32) $$\xi_{\mathrm{mn}}=\left\{\left\{x^{\prime },y^{\prime },t\right\}:\left\{x^{\prime },y^{\prime },0\right\}\in C^{\prime },m=x^{\prime },n=y^{\prime }\right\}.$$

The above formula represents the event trajectory, which signifies a set of events along the time axis projected onto pixel (m,n) after applying the warp field Φ. We define the event count image as follows:

(33) $$\Upsilon_{\mathrm{mn}}=\left|\xi_{\mathrm{mn}}\right|.$$

The formula for calculating the cell compensation event density D is as follows:

(34) $$D=\frac{\left|C^{\prime }\right|}{\Upsilon_{C}\cdot h_{C}\left(z,p\right)},$$

where $\Upsilon_{C}$ represents the number of pixels within cell C that have at least one event mapped to them. However, during the projection operation, events generated by different subject edges may be projected onto the same pixel, counteracting our goal of reducing interfering events. To alleviate this issue, we refer to the approach in [53], utilizing a time representation image derived from timestamp information to aid in the primary compensation process. The expression is as follows:

(35) $$\Psi_{\mathrm{mn}}=\frac{1}{\Upsilon_{\mathrm{mn}}}\sum \;t:t\in \xi_{\mathrm{mn}}.$$

Ψ is a discrete plane where each pixel contains the event timestamps mapped to it by the warp field Φ. By calculating the average value of the timestamps, we can use all available events to improve the fidelity of the main event stream. The method in [53] considers only the latest timestamp, but since the signal-to-noise ratio of events depends on the average illumination, this method does not perform well under low light conditions. Additionally, Ψ follows the three-dimensional structure of the event cloud, and its gradient provides a global measure of error in the primary compensation process, which can be minimized as follows:

(36) $$E=\sum \;∥G\left[m,n\right]∥=\sum \;\left(G_{x}^{2}\left[m,n\right]+G_{y}^{2}\left[m,n\right]\right),$$

where $G_{x}\left[m,n\right]$ and $G_{y}\left[m,n\right]$ represent the local spatial gradients of Ψ along the X and Y axes, respectively, calculated using the Sobel operator. Given that Formula (36) considers the global error of the event cloud within the warp field, we can decompose it into the following equations, assuming rigid body motion:

(37) $$g_{x}=\frac{\sum {\;G}_{x}\left[m,n\right]}{\Upsilon_{C}\cdot h_{C}\left(z,p\right)},$$

(38) $$g_{y}=\frac{\sum {\;G}_{y}\left[m,n\right]}{\Upsilon_{C}\cdot h_{C}\left(z,p\right)},$$

(39) $$g_{t}=\frac{\sum \;\left(G_{x}\left[m,n\right],G_{y}\left[m,n\right]\right)\cdot \left(m,n\right)}{\Upsilon_{C}\cdot h_{C}\left(z,p\right)},$$

(40) $$g_{\theta }=\frac{\sum \;\left(G_{x}\left[m,n\right],G_{y}\left[m,n\right]\right)\times \left(m,n\right)}{\Upsilon_{C}\cdot h_{C}\left(z,p\right)}.$$

The above four equations correspond to the local errors $\left\{E_{x},E_{y},E_{z},\theta \right\}$ in the displacement, scaling, and 2D rotation compensation processes on the image plane. We use a global main event model ${ℳ}^{G}=\left\{E_{x},E_{y},E_{z},\theta \right\}$ containing error parameters to describe the corresponding global warp field $\Phi^{G}\left(x,y,t\right)$, yielding the transformation as follows:

(41) $$\left[\begin{array}{l} x^{\prime }\\ y^{\prime } \end{array}\right]=\left[\begin{array}{l} x\\ y \end{array}\right]-t\;*\;\left[\left[\begin{array}{l} E_{x}\\ E_{y} \end{array}\right]+\left(E_{t}+1\right)\;*\;\left|\begin{array}{cc} \cos\theta & -\sin\theta \\ \sin\theta & \cos\theta \end{array}\right|\cdot \left[\begin{array}{l} x\\ y \end{array}\right]-\left[\begin{array}{l} x\\ y \end{array}\right]\right].$$

This transformation converts the coordinates {x,y,t} in the event stream to $\left\{x^{\prime },y^{\prime },t\right\}$, resulting in the expression of the warp field, with timestamps remaining unchanged. Based on the proposed four-parameter model, we estimate and minimize the motion range of the main event stream using an error function defined on the time representation image. By maximizing the main event density on the event count image, we refine the global activity area of the main events and identify and track a subset of non-main events with a high probability of correlation to the main events, thus achieving main event stream completion and motion compensation.

The entire main event completion process involves first using Formula (41) to calculate the initial warp field expression for each cell C, then using Formula (35) to generate the corresponding time representation image Ψ. We compute the model ${ℳ}^{G}$ and its corresponding gradient using the initial model ${ℳ}_{0}^{G}$ and Formulas (37)–(40) and update the range of the warp field of ${ℳ}^{G}$ through gradient descent, thus updating the region of the primary compensation events $C^{\prime }$. Next, we maximize the main event density D on the event count image Υ, refining the global activity area of the main events and further adjusting the region of the primary compensation events $C^{\prime }$. It is noteworthy that Υ and Ψ both represent local measures of the deviation between primary and non-main event regions in the event stream but are based on different data sources: event rate and event timestamps. The detailed process is as Algorithm 2, where the discrete parameter k represents the event camera pixel size of 0.3, q is an adjustable precision parameter, and D represents the initial value of the cell compensation event density, set to 0.
**Algorithm 2. Adaptive Spatiotemporal Subject Surface Compensation.**$\mathrm{Input}:\;\mathrm{Initial}\;\mathrm{model}\;{ℳ}_{0}^{G}$ and initial cell C of the main events, k, q **Output**: Cell $C^{\prime }$ and density D of main compensation events 1    Obtain main compensation events $C^{\prime }$ based on Formula (1)$,\;{ℳ}_{0}^{G},$ and C 2    Obtain time representation image Ψ based on Formula (5) $,C^{\prime },$ and k $3\;\;\;\;\mathrm{Obtain}\;{ℳ}^{G}$ based on Formulas (7–9)$,\;{ℳ}_{0}^{G},$ and Ψ $4\;\;\;\;\mathrm{While}\;{∥{ℳ}^{G}-{ℳ}_{0}^{G}∥}_{2}>q$ 5        Update main compensation events $C^{\prime }$ based on Formula (1)$,\;{ℳ}_{0}^{G},$ and C 6        Update time representation image Ψ based on Formula (5)$,\;C^{\prime },$ and k $7\;\;\;\;\;\;\;\;\;\mathrm{Update}\;\mathrm{model}\;{ℳ}^{G}$ based on Formulas (7–9)$,\;{ℳ}^{G},$ and Ψ 8    **End** 9    Obtain event count image Υbased on Formula (3)$,\;C^{\prime },$ and k 10   Obtain event density $D^{\prime }$ based on Formula (4) and Υ $11\;\;\;\mathrm{While}\;∥D^{\prime }-D∥>q$ 12        Assign the value of D to $D^{\prime }$ 13        Obtain event density D based on Formula (4), C, k,$\;\mathrm{and}\;{ℳ}^{G}$ 14        $\mathrm{If}\;D^{\prime }<D$
**do** 15           $\mathrm{Update}\;{ℳ}^{G}$ and $C^{\prime }$ 16           Update event count image Υ based on Formula (3)$,\;C^{\prime },$ and k 17        **End** 18   **End** 19   Return $C^{\prime }$, D


After obtaining the area range $C^{\prime }$ and the final event density D of the primary compensation events, we can determine the range of events in the original event stream that can supplement the main events in the new event stream. In this way, we further reduce the impact of non-main events with interfering effects in the new event stream and compensate for any main events that might have been removed in the previous section. Finally, by projecting the events within each event cuboid’s time span into the pixel plane in the form of an event count image, we complete the entire event stream representation process. Next, we need to quantitatively evaluate the processing results and compare the effectiveness of our algorithm with existing mainstream and latest event representation methods.

### 2.3. Actual Performance Efficiency Discrepancy

To evaluate the distortion degree of the final results obtained by various representation methods, we refer to the prior knowledge on event representation discrepancies in [52] and propose a new metric called Gromov–Wasserstein Event Discrepancy (GWED) based on events. For comparison purposes, we project the event streams processed by each method onto a plane using event count images. This approach ensures that each event stream possesses features indexed by the horizontal position of integer-valued pixels in the count image Υ, expressed as follows:

(42) $$f_{x}=\Upsilon \left(x\right)\in R^{N_{f}},$$

(43) $$ℱ={\left\{f_{x}\right\}}_{x\in \Lambda }.$$

Here, Λ represents the image domain, and $\left|\Lambda \right|=N_{f}$ represents the number of pixels in the image domain. ℱ denotes the set of features for each group of positions. When converting the original event representation, some important event features are inevitably removed from the event stream, leading to distortion. Therefore, we measure the actual damage to the event stream by examining the relationship between the feature set and the event stream.

**Figure 5:**
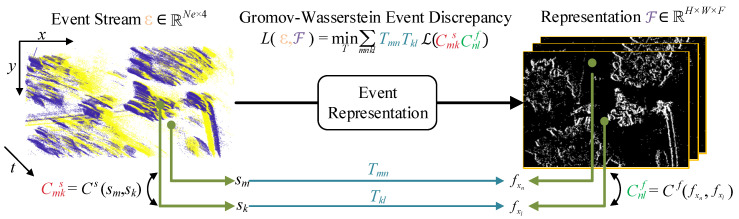
Schematic of the Gromov–Wasserstein Event Discrepancy between the original event stream and the event representation results.

Figure 5 provides an overview schematic of GWED. We first measure the similarity between each set of events and their corresponding representations by constructing a soft correspondence between event $s_{m}$ and the corresponding feature $f_{x_{n}}$, defining it as $T_{\mathrm{mn}}$. This transport relationship effectively captures the features corresponding to each event and identifies the distortion and destruction information of the original event set. We set the total weight of the events to 1, meaning each event has a weight of $1/N_{e}$. These event weights are transferred to the output features in the transformed representation, which also need a total weight of 1. Thus, each feature has a weight of $T_{\mathrm{mn}}$. To satisfy this construction, the corresponding equations $\sum_{m}T_{\mathrm{mn}}=1/N_{f}$ and $\sum_{n}T_{\mathrm{mn}}=1/N_{e}$ must hold true.

In the next step, we evaluate the distortion introduced in the aforementioned transport relationship by using the pairwise similarity of events and features, which reflects the damage to actual events during the event representation process, thereby obtaining the corresponding distortion rate. Let the features corresponding to a pair of events, $s_{m}$ and $s_{k},$ be $f_{x_{n}}$ and $f_{x_{l}}$, respectively. Their similarity ratings are as follows:

(44) $$C_{\mathrm{mk}}^{s}=C^{s}\left(s_{m},s_{k}\right),$$

(45) $$C_{\mathrm{nl}}^{f}=C^{f}\left(f_{x_{n}},f_{x_{l}}\right).$$

Then, using the difference in the previously proposed similarity scores as a measure of distortion, we define the metric for each pair of events and features as follows:

(46) $$L_{\mathrm{mnkl}}=T_{\mathrm{mn}}T_{\mathrm{kl}}ℒ\left(C_{\mathrm{mk}}^{s},C_{\mathrm{nl}}^{f}\right),$$

where ℒ represents the difference measurement between $C^{s}\left(s_{m},s_{k}\right)$ and $C_{\mathrm{nl}}^{f}$. In this process, we use the Gaussian Radial Basis Function (RBF) [54] and Kullback–Leibler (KL) divergence as the basis for calculating features and similarities related to events and images. The calculation process is as follows:

(47) $$C_{\mathrm{mk}}^{s}=e^{\frac{-{∥s_{m}-s_{k}∥}^{2}}{2\sigma_{s}^{2}}},$$

(48) $$C_{\mathrm{nl}}^{f}=e^{\frac{-{∥f_{x_{n}}-f_{x_{1}}∥}^{2}}{2\sigma_{f}^{2}}},$$

(49) $$\sigma_{s}^{2}={\mathrm{mean}}_{m<n}{∥s_{m}-s_{n}∥}^{2},$$

(50) $$\sigma_{f}^{2}={\mathrm{mean}}_{m<n}{∥f_{x_{m}}-f_{x_{n}}∥}^{2},$$

(51) $$ℒ\left(C_{\mathrm{mk}}^{s},C_{\mathrm{nl}}^{f}\right)=C_{\mathrm{mk}}^{s}\log\left(C_{\mathrm{mk}}^{s}/C_{\mathrm{nl}}^{f}\right).$$

By normalizing the distances between events and feature pairs with the variance of the event stream data, we ensure that the similarity scores are robust to data dimensions and the number of samples in the source and target domains. Summing over all possible pairs of events and features gives us the transport cost during the event representation process.

(52) $$L\left(T;ℰ,ℱ\right)=\sum_{m,n,k,l}L_{\mathrm{mnkl}}=\sum_{m,n,k,l}T_{\mathrm{mn}}T_{\mathrm{kl}}ℒ\left(C_{\mathrm{mk}}^{s},C_{\mathrm{nl}}^{f}\right).$$

Next, we minimize the transport relationship T to obtain the GWED as follows:

(53) $$\mathrm{GWED}=L\left(ℰ,ℱ\right)=\underset{T}{\min}\sum_{m,n,k,l}T_{\mathrm{mn}}T_{\mathrm{kl}}ℒ\left(C_{\mathrm{mk}}^{s},C_{\mathrm{nl}}^{f}\right).$$

Referring to the approach in [55], since the above metric is defined based on the events within a single event cuboid, we can average it over multiple samples to obtain ${\mathrm{GWED}}_{N}$ as follows:

(54) $${\mathrm{GWED}}_{N}=\frac{1}{N}\sum_{m}L\left({ℰ}_{m},{ℱ}_{m}\right),$$

**Figure 6:**
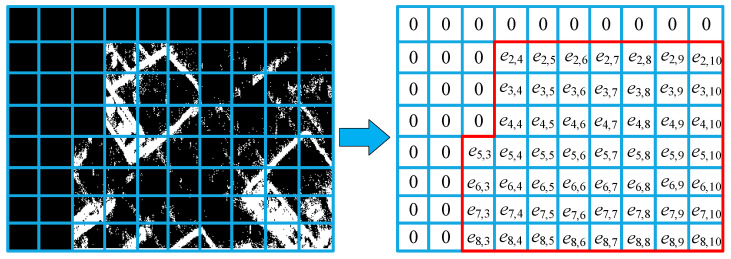
Illustration of the grid positions corresponding to non-zero entropy values.

Which represents the average distortion rate from the original event stream to the event representation process. We then refer to [35] to use confidence interval-related knowledge to calculate the retention rate of main events in the final distorted result. In this work, we use Non-Zero Grid Entropy (NZGE) as the metric. To calculate the NZGE value for the main event area in the generated count image, we first count the area corresponding to main events within a single event cuboid and divide this area into m×n cells of size C as before. Then, we calculate an image entropy for each cell to construct an entropy map, as shown in Figure 6.

The calculation process for the NZGE value within each event cuboid is as follows:

(55) $${\mathrm{NZGE}}_{i}=\frac{1}{n_{i}^{C}}\sum_{x=1}^{m}\sum_{y=1}^{n}{{\mathrm{entropy}}^{i}}_{x,y},$$

(56) $${\mathrm{entropy}}_{x,y}=-\sum_{z=0}^{255}p_{z}^{x,y}{\log\;p}_{z}^{x,y}.$$

$n_{i}^{C}$ represents the number of cells with non-zero entropy. ${{\mathrm{entropy}}^{i}}_{x,y}$ represents the image entropy of the region at the x row and y column. $p_{z}^{x,y}$ represents the probability that a pixel in the corresponding image region at the x row and y column has a grayscale value of z, with the grayscale range being 0 to 255. We can use the NZGE value to keep the primary contour part of the final result within a reasonable range, which requires a corresponding confidence interval. To calculate the upper and lower bounds of the confidence interval, let the collection of nn NZGE values be N, with the corresponding mean and variance being $\bar{N}$ and $S_{N}^{2}$. Assuming the appropriate NZGE values are independent and normally distributed, the set of observations for the normal distribution is N. Here, we define a pivotal quantity g as follows:

(57) $$g=\frac{\bar{N}-\mu }{S_{N}/\sqrt{n}}.$$

As a result, g follows the t-distribution t(n−1) with n−1 degrees of freedom. We can calculate the confidence interval for the NZGE value as follows:

(58) $$\left[\alpha ,\beta \right]=\left[\bar{N}-\left|g_{0.025}\right|\frac{S_{N}}{\sqrt{n}},\bar{N}+\left|g_{0.025}\right|\frac{S_{N}}{\sqrt{n}}\right].$$

When the NZGE value lies within the confidence interval, the primary information in the event representation image obtained from the corresponding event cuboid is well-preserved. The closer the NZGE value is to the median, the better the preservation. Therefore, we define the difference between the NZGE and the median as ${\mathrm{NZGE}}_{D}$:

(59) $${\mathrm{NZGE}}_{D}=\left|\mathrm{NZGE}-\mu \right|.$$

The smaller this value, the better the corresponding representation method preserves the main events. Combining this with the previously obtained ${\mathrm{GWED}}_{N}$, we obtain the new metric for retention efficiency discrepancy:

(60) $$\mathrm{APED}={\mathrm{GWED}}_{N}\cdot {\mathrm{NZGE}}_{D}.$$

This value represents the treatment strategies for actual events and main events in the event stream by various methods. The lower this metric, the lower the actual distortion rate of the corresponding event representation method, and the higher the retention rate of main events carrying major information.

## 3. Results

**Figure 7:**
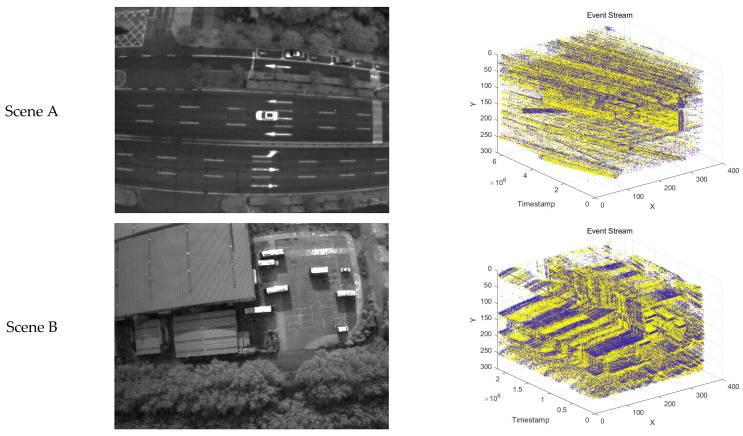

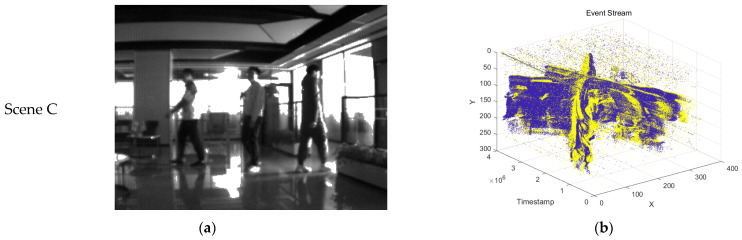
Grayscale images and 3D event stream diagrams for three captured scenarios: (**a**) Grayscale illustration of the corresponding scenarios; (**b**) 3D event stream illustration of the corresponding scenarios.

During the experimental phase, we used the DAVIS346 event camera to capture and establish a dataset for comparative experiments in various motion scenarios. The performance of this camera is sufficient to meet our needs for image acquisition and metric computation in complex motion scenes. For the experiments, we selected three scenarios to capture and compare the performance of different algorithms. These include two complex scenes with stationary objects and moving cameras, as well as one scene with multiple subjects moving at different speeds relative to the camera. The grayscale images and 3D event stream diagrams of these three scenarios are shown in Figure 7.

The algorithms involved in the experiments were implemented on the MATLAB 2022b platform. The computer used to run the algorithm programs was equipped with an Intel(R) Core(TM) i7-11800H @ 2.30 GHz processor, 16 GB of RAM, and an NVIDIA GeForce RTX 3060 6 GB GPU. The operating system was Windows 11. Before conducting the experiments, we chose to extract the events within the first 5000 ms of each event stream for comparative analysis. This is because the value of ${\mathrm{GWED}}_{N}$ in the event representation process can exhibit significant fluctuations when the number of event samples is small.

**Figure 8:**
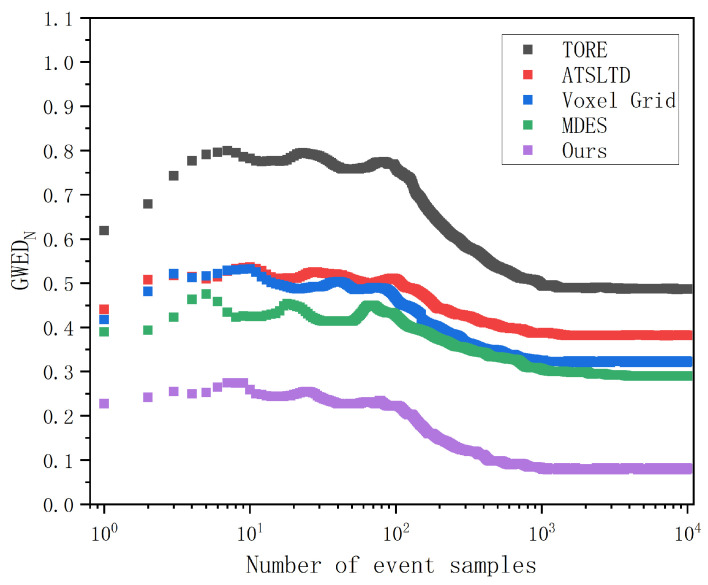
The variation of the value of ${\mathrm{GWED}}_{N}$ corresponding to each algorithm with different numbers of event samples.

Figure 8 shows the variation of the value of ${\mathrm{GWED}}_{N}$ corresponding to each algorithm with different numbers of event samples. In the comparison experiment for Scene A, as the number of samples increases, the value of ${\mathrm{GWED}}_{N}$ for each algorithm changes. Therefore, we extracted a 5000 ms segment from the original event streams of each scene as the basis for our experiments, randomly selecting 100,000 events for calculating this metric. This ensures that the value of ${\mathrm{GWED}}_{N}$ falls within a stable range that accurately reflects the distortion rate for each algorithm, providing sufficient experimental data to validate the performance comparison results. After this value stabilizes, the lower the corresponding algorithm, the better the performance. The algorithms used for comparison are the widely-used Voxel Grid [25], TORE [28], MDES [29], and ATSLTD [35].

**Figure 9:**
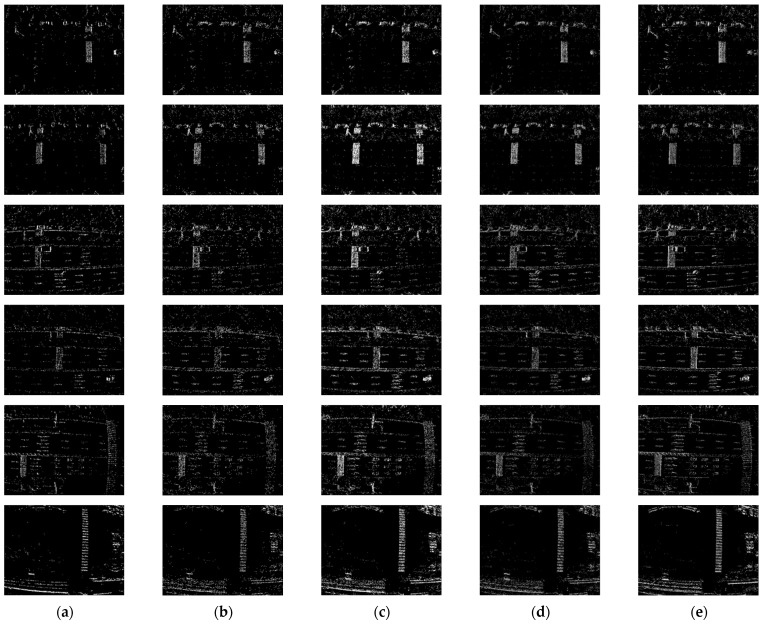
Illustration of the event stream processing results for Scene A by different algorithms: (**a**) TORE; (**b**) ATSLTD; (**c**) Voxel Grid; (**d**) MDES; (**e**) Ours.

**Figure 10:**
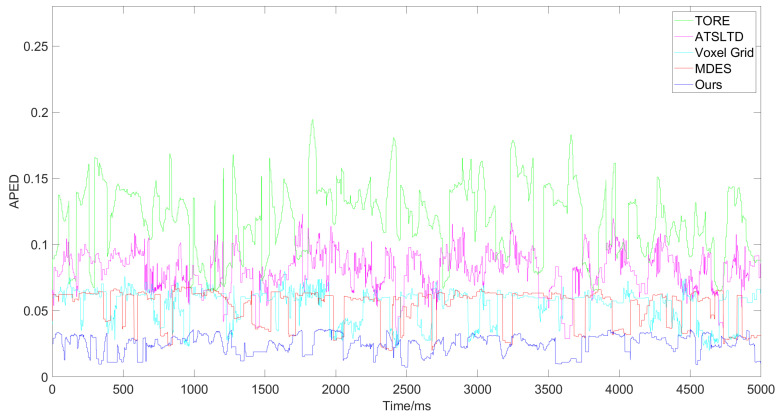
APED data obtained from the event stream processing results for Scene A by different algorithms.

**Figure 11:**
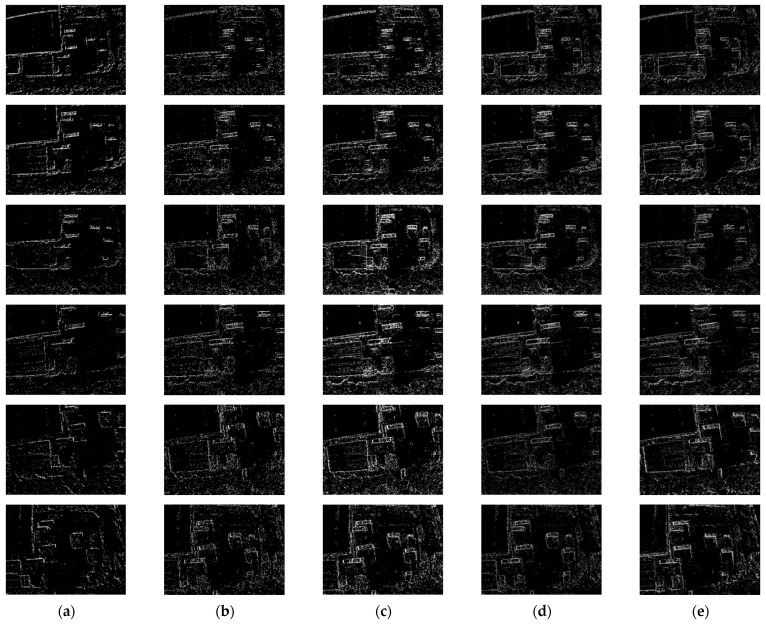
Illustration of the event stream processing results for Scene B by different algorithms: (**a**) TORE; (**b**) ATSLTD; (**c**) Voxel Grid; (**d**) MDES; (**e**) Ours.

**Figure 12:**
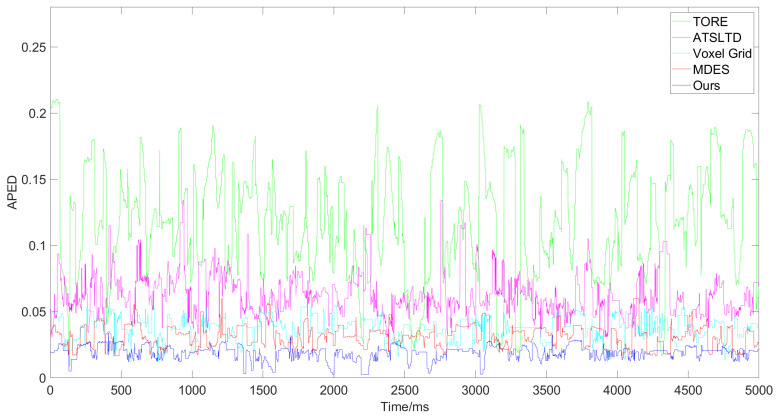
APED data obtained from the event stream processing results for Scene B by different algorithms.

First, we conducted comparative experiments on the event representation effectiveness of each algorithm for Scene A and Scene B. These scenes involve a drone equipped with an event camera capturing complex real-world environments while moving slowly at high altitudes. Figure 9 and Figure 10 show the results and corresponding APED values for each algorithm in Scene A, while Figure 11 and Figure 12 present the results for Scene B.

From the comparative results of the algorithms in these two scenes, it is evident that when the objects being captured have diverse compositions, complex textures, and numerous elements, the TORE and ATSLTD algorithms occasionally miss important information in the event presenting images. In contrast, the ATSLTD and Voxel Grid algorithms sometimes produce overly redundant results, failing to reflect textures and directional information. Our method, however, produces event-presenting images that fully capture the information of the objects without motion blur.

**Figure 13:**
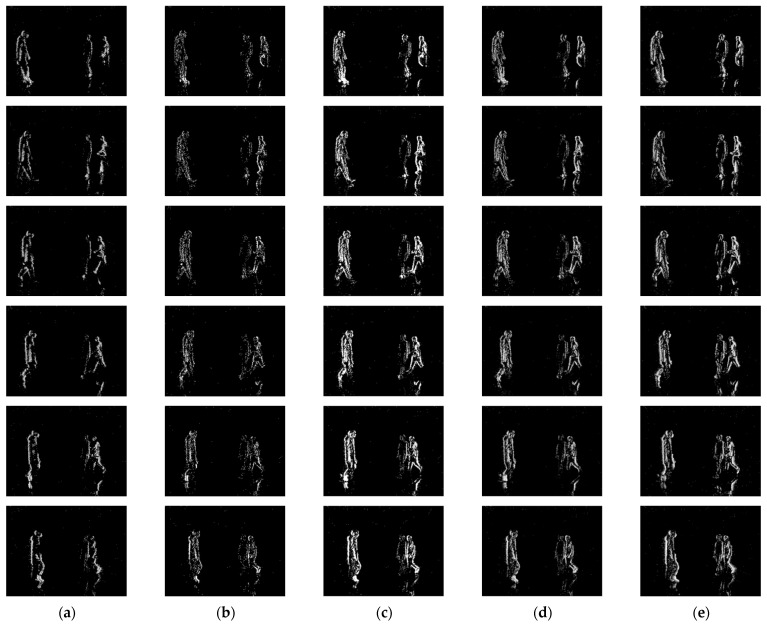
Illustration of the event stream processing results for Scene C by different algorithms: (**a**) TORE; (**b**) ATSLTD; (**c**) Voxel Grid; (**d**) MDES; (**e**) Ours.

**Figure 14:**
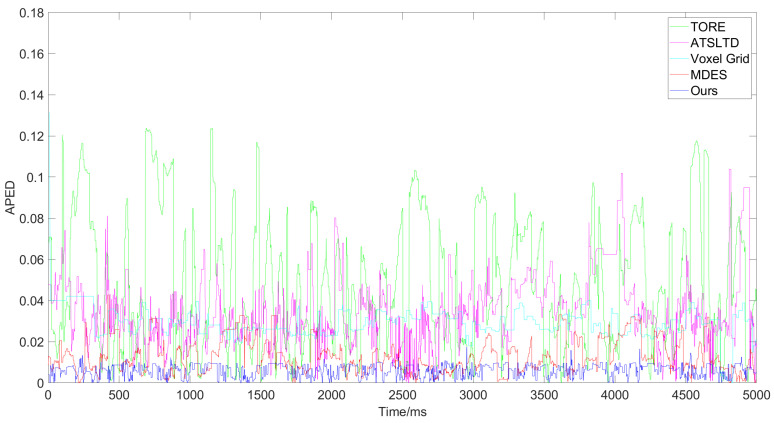
APED data obtained from the event stream processing results for Scene C by different algorithms.

Next, Figure 13 and Figure 14 show the results and corresponding APED values for each algorithm in Scene C, where there are multiple subjects moving at different speeds relative to the camera.

From Figure 13, it is clear that when there are multiple subjects moving at different speeds in the scene, the existing mainstream algorithms cannot fully represent the information of all subjects. Specifically, in Scene C, where three people are walking with the middle person moving significantly slower than the other two, the other algorithms used in the comparison experiment tend to lose part of the slower subject’s contour and texture information while ensuring the completeness of the other two subjects’ information. Our algorithm, on the other hand, can retain the information of all subjects, including the slower one, resulting in higher completeness of the slower subject’s information in the final event representation image.

Finally, we summarize the average APED values of each algorithm across the three comparison experiment scenes in Table 1.

Based on the data in this table, we can quantitatively evaluate the overall performance of each algorithm. It is evident that our algorithm significantly outperforms others in avoiding data distortion and redundancy while better preserving the main events that carry the main information.

## 4. Discussion

The experiments presented in this paper demonstrate that our event representation algorithm maintains excellent overall performance and effective representation when handling varying complexities and multiple subjects. In processing the two components of the APED metric, GWED_N_ and ${\mathrm{NZGE}}_{D}$, we observed that the former could not reliably reflect the performance and distortion rate of each algorithm with smaller sample sizes. Therefore, we selected a large random sample size to obtain stable metric values. For the latter component, we utilized a continuous segment of event stream data as an experimental basis, recognizing that event cameras encounter many unpredictable situations during actual operation. The ability to handle these scenarios needs to be incorporated into the comprehensive evaluation.

The demonstration images produced by various algorithms also indicate that under complex motion conditions or frequently changing scenes, some algorithms exhibit motion blur, contour dragging, ghosting, or information loss. In contrast, our algorithm maintains the integrity of scene information without redundancy across various scenarios. Additionally, experiments corresponding to Scene C reveal that when subjects move at significantly different speeds, most current algorithms cannot fully preserve the information completeness of all subjects. They often sacrifice information from slower-moving subjects to ensure a better representation of faster-moving ones. Our algorithm optimizes overall representation by compensating for the information of slower-moving subjects while preserving the performance of faster-moving subjects. The final APED value statistics demonstrate that our algorithm excels in both distortion minimization and main event retention.

## 5. Conclusions

This paper introduces a novel event data measurement and slicing algorithm, along with an event data completion and optimization algorithm, addressing the challenges and limitations of existing event representation methods when processing event stream data. Our approach preserves information completeness in complex scenes with diverse elements while minimizing redundant events and reducing motion blur, ghosting, and contour dragging. Additionally, it resolves the issue of maintaining information completeness for subjects moving at different speeds within a scene. Current event representation methods use differences in movement speeds between subjects to intuitively present speed-related information. However, when applying these representations to recognition and detection tasks, these speed differences can hinder further experiments. This issue does not arise with traditional images, where motion blur from high-speed movement can impede advanced experiments. Our algorithm combines the strengths of event cameras, which excel in capturing dynamic objects, with those of traditional cameras, which are better at capturing static objects. It ensures the completeness of high-speed object information without redundancy while fully representing the information of slower-moving objects through compensation. This approach reduces errors in recognition tasks caused by significant differences in individual movement speeds. Future research will focus on better complementing the missing information in event images with traditional images or integrating the two to achieve a more comprehensive representation, thereby attaining full environmental perception.

## Figures and Tables

**Table 1:** APED values of event stream data processed by different event representation algorithms in three captured scenarios.

Method	Actual Performance Efficiency Discrepancy		
	Scene A	Scene B	Scene C
**TORE**	0.11575	0.11906	0.04819
**ATSLTD**	0.07921	0.06497	0.03415
**Voxel Grid**	0.05566	0.03737	0.02832
**MDES**	0.05356	0.03086	0.01336
**Ours**	0.02403	0.01896	0.00596

## Data Availability

Data are contained within the article.

## References

[1] Lichtsteiner P., Posch C., Delbruck T. (2008). A 128 × 128 120 dB 15 μs latency asynchronous temporal contrast vision sensor. IEEE J. Solid-State Circuits.

[2] Brandli C., Berner R., Yang M., Liu S.-C., Delbruck T. (2014). A 240 × 180 130 db 3 µs latency global shutter spatiotemporal vision sensor. IEEE J. Solid-State Circuits.

[3] Posch C., Matolin D., Wohlgenannt R. (2010). A QVGA 143 dB dynamic range frame-free PWM image sensor with lossless pixel-level video compression and time-domain CDS. IEEE J. Solid-State Circuits.

[4] Oyster C. (1968). The analysis of image motion by the rabbit retina. J. Physiol..

[5] Murphy-Baum B.L., Awatramani G.B. (2018). An old neuron learns new tricks: Redefining motion processing in the primate retina. Neuron.

[6] Ölveczky B.P., Baccus S.A., Meister M. (2003). Segregation of object and background motion in the retina. Nature.

[7] Wild B. (2018). How does the brain tell self-motion from object motion?. J. Neurosci..

[8] Ghosh R., Gupta A., Nakagawa A., Soares A., Thakor N. (2019). Spatiotemporal filtering for event-based action recognition. arXiv.

[9] Ghosh R., Gupta A., Tang S., Soares A., Thakor N. (2019). Spatiotemporal feature learning for event-based vision. arXiv.

[10] Orchard G., Meyer C., Etienne-Cummings R., Posch C., Thakor N., Benosman R. (2015). HFirst: A temporal approach to object recognition. IEEE Trans. Pattern Anal. Mach. Intell..

[11] Lee J.H., Delbruck T., Pfeiffer M. (2016). Training deep spiking neural networks using backpropagation. Front. Neurosci..

[12] Zhao B., Ding R., Chen S., Linares-Barranco B., Tang H. (2014). Feedforward categorization on AER motion events using cortex-like features in a spiking neural network. IEEE Trans. Neural Netw. Learn. Syst..

[13] Pérez-Carrasco J.A., Zhao B., Serrano C., Acha B., Serrano-Gotarredona T., Chen S., Linares-Barranco B. (2013). Mapping from frame-driven to frame-free event-driven vision systems by low-rate rate coding and coincidence processing—Application to feedforward ConvNets. IEEE Trans. Pattern Anal. Mach. Intell..

[14] Sekikawa Y., Hara K., Saito H. Eventnet: Asynchronous recursive event processing. Proceedings of the IEEE/CVF Conference on Computer Vision and Pattern Recognition.

[15] Qi C.R., Yi L., Su H., Guibas L.J. (2017). Pointnet++: Deep hierarchical feature learning on point sets in a metric space. Adv. Neural Inf. Process. Syst..

[16] Fan H., Yu X., Ding Y., Yang Y., Kankanhalli M. (2022). Pstnet: Point spatio-temporal convolution on point cloud sequences. arXiv.

[17] Gehrig M., Scaramuzza D. Recurrent vision transformers for object detection with event cameras. Proceedings of the IEEE/CVF Conference on Computer Vision and Pattern Recognition.

[18] Schaefer S., Gehrig D., Scaramuzza D. Aegnn: Asynchronous event-based graph neural networks. Proceedings of the IEEE/CVF Conference on Computer Vision and Pattern Recognition.

[19] Bi Y., Chadha A., Abbas A., Bourtsoulatze E., Andreopoulos Y. (2020). Graph-based spatio-temporal feature learning for neuromorphic vision sensing. IEEE Trans. Image Process..

[20] Bi Y., Chadha A., Abbas A., Bourtsoulatze E., Andreopoulos Y. Graph-based object classification for neuromorphic vision sensing. Proceedings of the IEEE/CVF International Conference on Computer Vision.

[21] Mondal A., Giraldo J.H., Bouwmans T., Chowdhury A.S. Moving object detection for event-based vision using graph spectral clustering. Proceedings of the IEEE/CVF International Conference on Computer Vision.

[22] Deng Y., Chen H., Liu H., Li Y. A voxel graph cnn for object classification with event cameras. Proceedings of the IEEE/CVF Conference on Computer Vision and Pattern Recognition.

[23] Maqueda A.I., Loquercio A., Gallego G., García N., Scaramuzza D. Event-based vision meets deep learning on steering prediction for self-driving cars. Proceedings of the IEEE Conference on Computer Vision and Pattern Recognition.

[24] Sironi A., Brambilla M., Bourdis N., Lagorce X., Benosman R. HATS: Histograms of averaged time surfaces for robust event-based object classification. Proceedings of the IEEE Conference on Computer Vision and Pattern Recognition.

[25] Zhu A.Z., Yuan L., Chaney K., Daniilidis K. Unsupervised event-based learning of optical flow, depth, and egomotion. Proceedings of the IEEE/CVF Conference on Computer Vision and Pattern Recognition.

[26] Wang L., Ho Y.-S., Yoon K.-J. Event-based high dynamic range image and very high frame rate video generation using conditional generative adversarial networks. Proceedings of the IEEE/CVF Conference on Computer Vision and Pattern Recognition.

[27] Alonso I., Murillo A.C. EV-SegNet: Semantic segmentation for event-based cameras. Proceedings of the IEEE/CVF Conference on Computer Vision and Pattern Recognition Workshops.

[28] Baldwin R.W., Liu R., Almatrafi M., Asari V., Hirakawa K. (2022). Time-ordered recent event (tore) volumes for event cameras. IEEE Trans. Pattern Anal. Mach. Intell..

[29] Nam Y., Mostafavi M., Yoon K.-J., Choi J. Stereo depth from events cameras: Concentrate and focus on the future. Proceedings of the IEEE/CVF Conference on Computer Vision and Pattern Recognition.

[30] Zhang Y., Zhao Y., Lv H., Feng Y., Liu H., Han C. (2022). Adaptive slicing method of the spatiotemporal event stream obtained from a dynamic vision sensor. Sensors.

[31] Perot E., De Tournemire P., Nitti D., Masci J., Sironi A. (2020). Learning to detect objects with a 1 megapixel event camera. Adv. Neural Inf. Process. Syst..

[32] Kim J., Bae J., Park G., Zhang D., Kim Y.M. N-imagenet: Towards robust, fine-grained object recognition with event cameras. Proceedings of the IEEE/CVF International Conference on Computer Vision.

[33] Gehrig D., Loquercio A., Derpanis K.G., Scaramuzza D. End-to-end learning of representations for asynchronous event-based data. Proceedings of the IEEE/CVF International Conference on Computer Vision.

[34] Zubic N., Gehrig D., Gehrig M., Scaramuzza D. From Chaos Comes Order: Ordering Event Representations for Object Detection. Proceedings of the IEEE/CVF International Conference on Computer Vision (ICCV).

[35] Chen H., Wu Q., Liang Y., Gao X., Wang H. Asynchronous tracking-by-detection on adaptive time surfaces for event-based object tracking. Proceedings of the 27th ACM International Conference on Multimedia.

[36] Tang S., Lv H., Zhao Y., Feng Y., Liu H., Bi G. (2023). Denoising method based on salient region recognition for the spatiotemporal event stream. Sensors.

[37] Park I.M., Seth S., Paiva A.R., Li L., Principe J.C. (2013). Kernel methods on spike train space for neuroscience: A tutorial. IEEE Signal Process. Mag..

[38] González J.A., Rodríguez-Cortés F.J., Cronie O., Mateu J. (2016). Spatio-temporal point process statistics: A review. Spat. Stat..

[39] Teixeira R.F., Leite N.J. (2016). A new framework for quality assessment of high-resolution fingerprint images. IEEE Trans. Pattern Anal. Mach. Intell..

[40] Dong S., Bi Z., Tian Y., Huang T. (2018). Spike coding for dynamic vision sensor in intelligent driving. IEEE Internet Things J..

[41] Paiva A.R., Park I., Principe J.C. (2009). A reproducing kernel Hilbert space framework for spike train signal processing. Neural Comput..

[42] Tezuka T. (2018). Multineuron spike train analysis with R-convolution linear combination kernel. Neural Netw..

[43] Houghton C., Sen K. (2008). A new multineuron spike train metric. Neural Comput..

[44] Brockmeier A.J., Choi J.S., Kriminger E.G., Francis J.T., Principe J.C. (2014). Neural decoding with kernel-based metric learning. Neural Comput..

[45] Li J., Li J., Zhu L., Xiang X., Huang T., Tian Y. (2022). Asynchronous spatio-temporal memory network for continuous event-based object detection. IEEE Trans. Image Process..

[46] Gönen M., Alpaydın E. (2011). Multiple kernel learning algorithms. J. Mach. Learn. Res..

[47] Fu Y., Li J., Dong S., Tian Y., Huang T. Spike coding: Towards lossy compression for dynamic vision sensor. Proceedings of the 2019 Data Compression Conference (DCC).

[48] Scheerlinck C., Barnes N., Mahony R. Continuous-time intensity estimation using event cameras. Proceedings of the Asian Conference on Computer Vision.

[49] Rebecq H., Ranftl R., Koltun V., Scaramuzza D. Events-to-video: Bringing modern computer vision to event cameras. Proceedings of the IEEE/CVF Conference on Computer Vision and Pattern Recognition.

[50] Lagorce X., Orchard G., Galluppi F., Shi B.E., Benosman R.B. (2016). Hots: A hierarchy of event-based time-surfaces for pattern recognition. IEEE Trans. Pattern Anal. Mach. Intell..

[51] Marchisio A., Shafique M. (2023). Embedded Neuromorphic Using Intel’s Loihi Processor. Embedded Machine Learning for Cyber-Physical, IoT, and Edge Computing: Software Optimizations and Hardware/Software Codesign.

[52] Dalal N., Triggs B. Histograms of oriented gradients for human detection. Proceedings of the 2005 IEEE Computer Society Conference on Computer Vision and Pattern Recognition (CVPR’05).

[53] Zhu A.Z., Yuan L., Chaney K., Daniilidis K. (2018). EV-FlowNet: Self-supervised optical flow estimation for event-based cameras. arXiv.

[54] Said S., Bombrun L., Berthoumieu Y., Manton J.H. (2017). Riemannian Gaussian distributions on the space of symmetric positive definite matrices. IEEE Trans. Inf. Theory.

[55] Peyré G., Cuturi M., Solomon J. Gromov-wasserstein averaging of kernel and distance matrices. Proceedings of the International Conference on Machine Learning.

